# Correlations between personality traits, personality disorders, and immunometabolic markers

**DOI:** 10.1038/s41598-024-62214-9

**Published:** 2024-05-21

**Authors:** Hanna Spangenberg, Mia Ramklint, Janet L. Cunningham, Adriana Ramirez

**Affiliations:** 1 Department of Medical Sciences, Child and Adolescent Psychiatry, Uppsala University, Uppsala, Sweden; 2 Department of Medical Sciences, Psychiatry, Uppsala University, Uppsala, Sweden

**Keywords:** Biomarkers, Human behaviour

## Abstract

Evidence links immune system alterations to major psychiatric disorders. The few previous studies on personality traits or personality disorders (PDs) indicate that immunometabolic dysregulation may be prevalent in this population. This study aimed to investigate relationships between personality traits, PDs, and immunometabolic markers in peripheral blood. We hypothesized that neuroticism would be correlated with elevated leptin. Participants were recruited as young adults seeking care for general psychiatric disorders. They responded to a personality inventory and were assessed for PDs, and reevaluated again at a 12 years follow-up. Blood samples were collected at the follow-up and analyzed for 29 immunometabolic markers. A positive correlation was found between the personality trait neuroticism and leptin (ρ = 0.31, *p* = 0.02). An exploratory analysis also revealed a positive correlation between brain-derived neurotrophic factor (ρ = 0.36, *p* < 0.01) and neuroticism. These findings remained after adjusting for other variables in general linear models. There were no relationships between PDs and any immunometabolic markers. Results both confirm previous findings of correlations between the immunometabolic system and personality traits and suggest directions for future research.

## Introduction

There is mounting evidence of links between major psychiatric disorders and alterations in the immune system of adults^1,2^. Research on links between inflammation and major psychiatric disorders in young adults is more limited, and the results are less consistent^3^. Furthermore, there are few long-term follow-up studies of immunologic alterations in young psychiatric patients.

Personality disorder (PD) commonly presents in young adulthood^4,5^. PDs are characterized by deviant personality traits which are stable over time and across situations^6^, leading to impaired function within several areas of life, such as relationships and labor market outcomes^7,8^. In the Diagnostic and Statistical Manual of Mental Disorders, 5th edition (DSM-5)^6^, PDs are classified as categorical constructs, strictly separating normal from abnormal personality. Normal personality, unlike the PD construct, is dimensional, with individuals placed on a continuous spectrum for each personality trait^9^. Personality traits of anxiety, aggression, and low socialization have been shown to be related to the presence of a PD^10^.

In line with the strong scientific support in favor of immunological underpinnings of major psychiatric disorders, there are findings on connections between immunological patterns and personality traits, although they are not as numerous. Neuroticism, as a trait of normal personality, has been shown to be associated with elevated pro-inflammatory markers, such as interleukin 6 (IL-6), in blood^11,12^, and the same has been suggested to apply for the trait extraversion^13,14^. The trait conscientiousness, on the other hand, has been linked to lower levels of IL-6 in blood^13,15^. Some studies have shown that patterns can differ between sexes^14^. Immunometabolic markers, such as the appetite-regulating hormone leptin, have also been shown to be correlated with traits of normal personality. One study showed that low conscientiousness, but not neuroticism, was associated with high leptin levels in blood^16^, whereas another showed a correlation between high leptin levels in blood and neuroticism^11^. However, studies on correlations between personality traits and immunometabolic markers are limited, especially studies that include temporal factors, making the current study important.

Leptin has emerged as a biomarker of interest in the study of biological underpinnings of psychiatric disorders such as depression, but further studies of its role in specific affective domains are called for^17–19^. The mechanism for how leptin might affect mood remains unclear. Preclinical studies suggest direct neuroactive effects and that altered leptin signaling resulting in functional leptin resistance may play a role^17^. Another metabolic marker of interest in personality research is hemoglobin A1c (HbA1c), which reflects glycemic status over the preceding three months. HbA1c has been shown to be lower in individuals with high conscientiousness^20^. Since leptin and HbA1c are metabolic markers, there is a growing interest for research on their relation to personality and PD. Neuroticism has been found to be associated with an increased risk of the metabolic syndrome^21,22^, but associations between the metabolic syndrome and PDs are unclear. However, there is a markedly increased mortality in PD^23^, and there is a growing interest for investigating whether metabolic complications might be a factor behind this.

Studies investigating inflammatory patterns in PDs show inconsistent results. Some studies indicate elevated levels of inflammatory markers, such as C-reactive protein (CRP), in individuals with PD^24,25^. Others show more ambiguous results^26^, and some report on inflammatory patterns for specific PDs, not covering the entire PD spectrum^27^. Tasci et al.^28^ showed lower levels of leptin in blood in subjects with antisocial PD and Atmaca et al.^29^ showed the same for borderline PD, but to the best of our knowledge no study has examined leptin levels from a pan-diagnostic PD perspective. Inconsistencies as well as gaps in previous findings for immunometabolic markers in PD call for extended research.

The aim of this study was to test the hypothesis that elevated leptin in blood is correlated with sustained neurotic traits as shown in our previous study of young adults^11^. We additionally performed an exploratory analysis of an expanded set of chemokines and growth factors in blood samples taken from a cohort of young former psychiatric patients, followed up after 12 years, focusing on correlations with personality traits or PDs.

## Material and methods

### Participants

This was a follow-up study of a cohort of participants included in a study in 2002–2003^30^. Participants in the original study were recruited from a psychiatric outpatient clinic in Uppsala, Sweden. All individuals aged 18–25 years (n = 217) who sought help at the outpatient clinic between September 2002 and August 2003 were asked about inclusion in the study, and 200 (92%) agreed. Participants were assessed in accordance with the Longitudinal evaluation (L), done by experts (E), all data available (AD)—LEAD diagnostic procedure^31^, including the Structured Clinical Interview for DSM-IV Axis I Disorders Clinical Version, SCID-I-CV^32^, and the Structured Clinical Interview for DSM-IV Personality Disorders, SCID-II^33^, interviews. In total, 188 participants underwent all the assessments.

In 2016, current addresses of the former participants (n = 200) were retrieved from the Swedish Tax Agency, and letters containing information about the follow-up study were sent out. Informed consent to participate was received from 103 (52%) participants, whereas two (1%) participants refused further participation and requested to be removed from the cohort. The follow-up study encompassed self-report instruments which participants filled out over the internet or as paper-and-pencil questionnaires. One meeting was held with each participant, at which psychiatric diagnostic assessments and a somatic assessment including drawing of blood samples were performed. Participants could take part in all assessments or choose to participate in just a few. Elements included in the assessments are described below and have also been described in a previous publication^34^.

A total of 57 participants donated blood samples. Individuals with systemic inflammatory disease (n = 2) or pregnancy (n = 1) were excluded from this study, leaving 54 (27%) participants from the original cohort eligible for the current study. Participants and those lost to follow-up are described in Table 1, which shows some minor differences between these two groups: follow-up participants had no substance abuse/dependence and described fewer extroverted personality traits.

**Table 1:** Descriptive baseline (2002–2003) data for cohort participants (n = 54) in 2016 and those lost to follow-up (n = 144).

	Participants	Lost to follow-up	*p*
Personal data	n = 54	n = 144	
Age, years, mean (SD)	22.5 (1.9)	22.4 (1.9)	0.53
Sex, F, n (%)	47 (87)	112 (78)	0.15
	n = 46	n = 112	
BMI, kg/m^2^, mean (SD)	22.1 (4.5)	22.5 (4.1)	0.66
SSP dimension	n = 51	n = 128	
SSP neuroticism mean score (SD)	61.6 (9.2)	60.3 (10.3)	0.42
SSP aggressiveness mean score (SD)	49.7 (5.8)	51.6 (5.6)	0.06
SSP extraversion mean score (SD)	45.8 (6.5)	49.3 (7.3)	0.002*
Current psychiatric disorder	n = 54	n = 144	
Any anxiety disorder, n (%)	32 (59)	102 (71)	0.12
Any mood disorder, n (%)	41 (76)	102 (71)	0.58
Any substance abuse/dependence, n (%)	0 (0)	16 (11)	0.01*
Any eating disorder, n (%)	12 (22)	42 (29)	0.33
Current PD diagnosis	n = 51	n = 135	
Any PD diagnosis, n (%)	16 (31)	36 (27)	0.52
SCID-II score, mean (SD)	108.6 (13.0)	109.6 (18.4)	0.70

*BMI* body mass index, *PD* personality disorder, *SCID-II* structured clinical interview for DSM-IV personality disorders, *SSP* Swedish Universities Scales of Personality.

*Significant difference, *p* < 0.05.

### Self-report instruments

Follow-up participants filled out information on background data such as somatic health, current medication, and psychosocial data, as well as eight self-report instruments, as has been described in a previous publication^34^. Data from two of the eight self-report instruments were analyzed for the current study, namely the Swedish Universities Scales of Personalities (SSP), and the Comprehensive Psychopathological Rating Scale—Self-rating for affective disorder (CPRS-S-A), which are described below.

#### Swedish Universities Scales of Personality (SSP)

The SSP evaluates personality traits correlating with psychopathology^35^. The instrument consists of 91 self-response items on 13 scales: somatic trait anxiety, psychic trait anxiety, stress susceptibility, lack of assertiveness, impulsiveness, adventure seeking, detachment, social desirability, embitterment, trait irritability, mistrust, verbal trait aggression, and physical trait aggression. The 13 scales can be classified into three personality dimensions: neuroticism, extraversion, and aggressiveness. The merging of the 13 scales into three dimensions is based on a factor analysis by the original authors of the instrument. The SSP has been shown to have good psychometric properties. In the original work, Cronbach’s alpha coefficients ranged from 0.59 to 0.84. In this study, SSP data are expressed as T-scores in relation to normative data from the general population taken from the validation study of the instrument, where a T-score of 50 was the median^35^.

#### Comprehensive psychopathological rating: self-rating for affective disorder (CPRS-S-A)

The CPRS-S-A rates symptoms of anxiety, depression, and compulsion corresponding to three subscales for affective and anxiety syndromes. The subscale which is derived from the items measuring depression is the well-known Montgomery-Asberg depression rating scale self-assessment^36^. The CPRS-S-A consists of 19 items which the respondent rates based on symptom severity during the preceding three days. Symptoms are rated on a seven-point scale ranging from 0 to 3, with higher scores indicating higher symptom severity.

### Psychiatric assessment

Assessments at follow-up consisted of a clinical interview, and SCID-I-CV and SCID-II interviews. The SCID-II screening questionnaire had been filled out by the participant prior to their SCID-II interview^37^. Only items which the participant had screened positively for were included the interview—other items were left out. Two psychiatrists, a psychiatric registrar, and a medical student in the final year of training conducted all psychiatric assessments. Assessors were trained in accordance with the SCID manual and had been tested for interrater reliability (IR) prior to assessments. The IRs for SCID-II interviews were all excellent, with the lowest kappa value between raters being 0.89.

### Somatic assessment

Somatic assessments consisted of measurement of weight, height, waist and hip circumference, pulse, and blood pressure. Weight was measured in kilograms (kg) using a digital scale. Height was measured in meters (m). Body mass index (BMI) (kg/m^2^) was calculated by dividing weight by height squared.

#### Blood samples

EDTA plasma was collected from participants during office hours and stored at -80 °C. Immunometabolic markers were measured using a commercially available assay, in accordance with the manufacturer’s protocol (MULTI-ARRAY Assay System, Meso Scale Discovery, Rockville, MD, USA). To detect a range of innate and adaptive immunological responses, metabolic inflammations, as well as early signs of vascular injury, we chose markers that have been informative in our earlier work^18,38,39^ and in unpublished data from pilot studies in other cohorts. The analysis was performed by an accredited laboratory (SciLife, Uppsala, Sweden). Electro-chemiluminescent signals corresponding to cytokine targets were detected using a SECTOR Imager instrument (Meso Scale, Rockville, MD, USA). The Meso Scale analyzing kits we used were Vascular Injury V-plex (K15198D), Cytokine V-plex (K1510H), Pro-inflammatory V-plex (K15049D), Chemokine V-plex (K15047D), and a custom assay (U-plex; RANTES, B-cell-activating factor, brain-derived neurotrophic factor (BDNF), beta nerve growth factor, interferon (IFN)-a2a, leptin, and stromal cell-derived factor 1a (SDF-1a)). All samples were analyzed at the same timepoint. Values below lowest level of detection (LLOD) were replaced with 1/10 of LLOD in further analyses for these markers. Whole EDTA blood was analyzed for HbA1C by the Department of Clinical Chemistry at Uppsala University Hospital using the automated instrument Cobas c501 (Roche Diagnostics). Reagents were purchased from Roche Diagnostics (Reagent Cobas Integra® Hba1c Gen2 D/S. art.nr. 04528123190, Roche Diagnostics, Bromma, Sweden) and analysis was conducted in accordance with the manufacturer’s instructions.

### Statistics

All data on biological markers were Z-transformed prior to analyses in order to obtain comparable data. Agreement between raters was calculated using Cohen’s kappa values for evenly distributed data and prevalence- and bias-adjusted kappa (PABAK) for unevenly distributed data^40^. Spearman’s correlation coefficients were calculated between immunometabolic markers and SSP neuroticism score, SSP extraversion score, and SSP aggressiveness score, as well as for CPRS-S-A scores. Correlations between immunometabolic markers and SSP score, SCID-II total score, and CPRS-S-A score were visualized on a heatmap. Generalized linear models (GLzMs), using gamma distribution with log link function, were used for analyses with the outcome being immunometabolic markers that showed correlation above 0.30 with SSP neuroticism score, SSP extraversion score, or SSP aggressiveness score. GLzMs were adjusted for BMI, current antidepressant treatment, and sex. A sensitivity analysis was run for data with males excluded. The Reliable Change Index (RCI) was used to analyze the magnitude of change in SSP between the time points. RCI was calculated as (X2-X1)/SEM (standard error of measurement). Changes >  ± 1.96 were considered as reliable.

Data analyses were conducted using IBM SPSS Statistics version 28. Statistical significance was set at *p* < 0.05 and two-tailed. Because of the exploratory approach of the statistical analyses, no correction for multiple testing was performed.

### Ethical considerations

The study was carried out in accordance with the Helsinki Declaration and was approved by the Regional Ethics Committee at Uppsala University (Reference No. 2012/081, 2015/302). Participants were given verbal and written information on the study prior to inclusion, and all gave written consent to participate.

## Results

### Participant characteristics

Seven (13%) participants were male and 47 (87%) were female. There were 19 (35%) participants with a PD diagnosis at assessment, of which two participants had cluster B PD, and 17 participants had cluster C PD. There was no participant with a cluster A PD. Use of antidepressant treatment (selective serotonin receptor inhibitors or serotonin and norepinephrine reuptake inhibitors) was reported by 22 (41%) participants. Three (6%) participants reported use of mirtazapine, five (9%) reported use of atypical antipsychotics, and two (4%) reported use of lithium. Two (4%) participants reported daily smoking. Participants’ CPRS-S-A scores were moderate, with a mean total score of 12.9 (8.1). Participants are further described in Table 2.

**Table 2:** Descriptive data on study participants (n = 54).

Personal data	Mean (range)
Age, years	35.6 (32–39)
BMI, kg/m^2^	25.5 (18.0–43.0)
SSP dimension	Mean (range)
Neuroticism	55.8 (34.5–76.3)
Aggressiveness	48.3 (34.5–61.1)
Extraversion	46.5 (32.2–64.8)
CPRS-S-A	
CPRS-S-A total score	12.9 (0–32)
CPRS-S-A depression score	6.2 (0–17.5)
Current psychiatric disorder (SCID-I-CV) (n = 53)	n (%)
Any anxiety disorder	27 (50.9)
Any mood disorder	12 (22.6)
Any substance-related disorder	2 (3.7)
Any eating disorder	6 (11.3)
Current PD diagnosis	
Any PD, n (%)	19 (35)
SCID-II score, mean (range)	60.1 (9–153)

*BMI* body mass index, *CPRS-S-A* Comprehensive Psychopathological Rating Scale – Self-rating for affective disorder, *PD* personality disorder, *SCID-I-CV* structured clinical interview for DSM-IV Axis I disorders clinical version, *SCID-II* structured clinical interview for DSM-IV personality disorders, *SSP* Swedish Universities Scales of Personality.

### Immunometabolic markers in correlation to personality traits and PD

There was no participant with CRP levels above the clinical threshold value (> 10 $\upmu$g/ml) or with HbA1c above the clinical threshold value for diabetes (42 mmol/mol). The distributions of immunometabolic markers in the sample, LLOD, numbers of samples above LLOD, and coefficients of variation are shown in Supplementary table S1.

Trait neuroticism and leptin was found to have a positive Spearman’s correlation (ρ = 0.31, *p* = 0.02). Regression analyses for leptin and neuroticism revealed an odds ratio (OR) of 1.06 (95% CI 1.01; 1.10, *p* < 0.01) in the crude model. When controlled for sex and current antidepressant treatment, the significant effect remained (OR 1.05, 95% CI 1.01; 1.09, *p* = 0.02), which on average corresponded to an increase in leptin of 506 pg/ml per unit increase in neuroticism score. BMI was not adjusted for, as it was highly correlated with leptin (ρ = 0.78) and was regarded as a cofactor rather than a confounder. However, leptin might act as a proxy for the association between BMI and neuroticism (ρ = 0.62, *p* < 0.001). Because levels of both leptin and neuroticism were higher in females, the data for females was reanalyzed excluding males, the OR for leptin and neuroticism in the crude model was 1.06 (95% CI 1.01; 1.10, *p* = 0.01). When adjusted for current antidepressant treatment, the effect remained (OR 1.06, 95% CI 1.01; 1.10, *p* = 0.01). This corresponded to an average increase in leptin of 647 pg/ml per unit increase in neuroticism score. The findings remained in a sensitivity analysis where cases who were smokers (n = 2) or on current treatment with mirtazapine (n = 3), atypical antipsychotics (n = 5), or lithium (n = 2) were excluded. Leptin was furthermore found to be correlated with HbA1c (ρ = 0.33, *p* = 0.02) in males and females.

**Figure 1:**
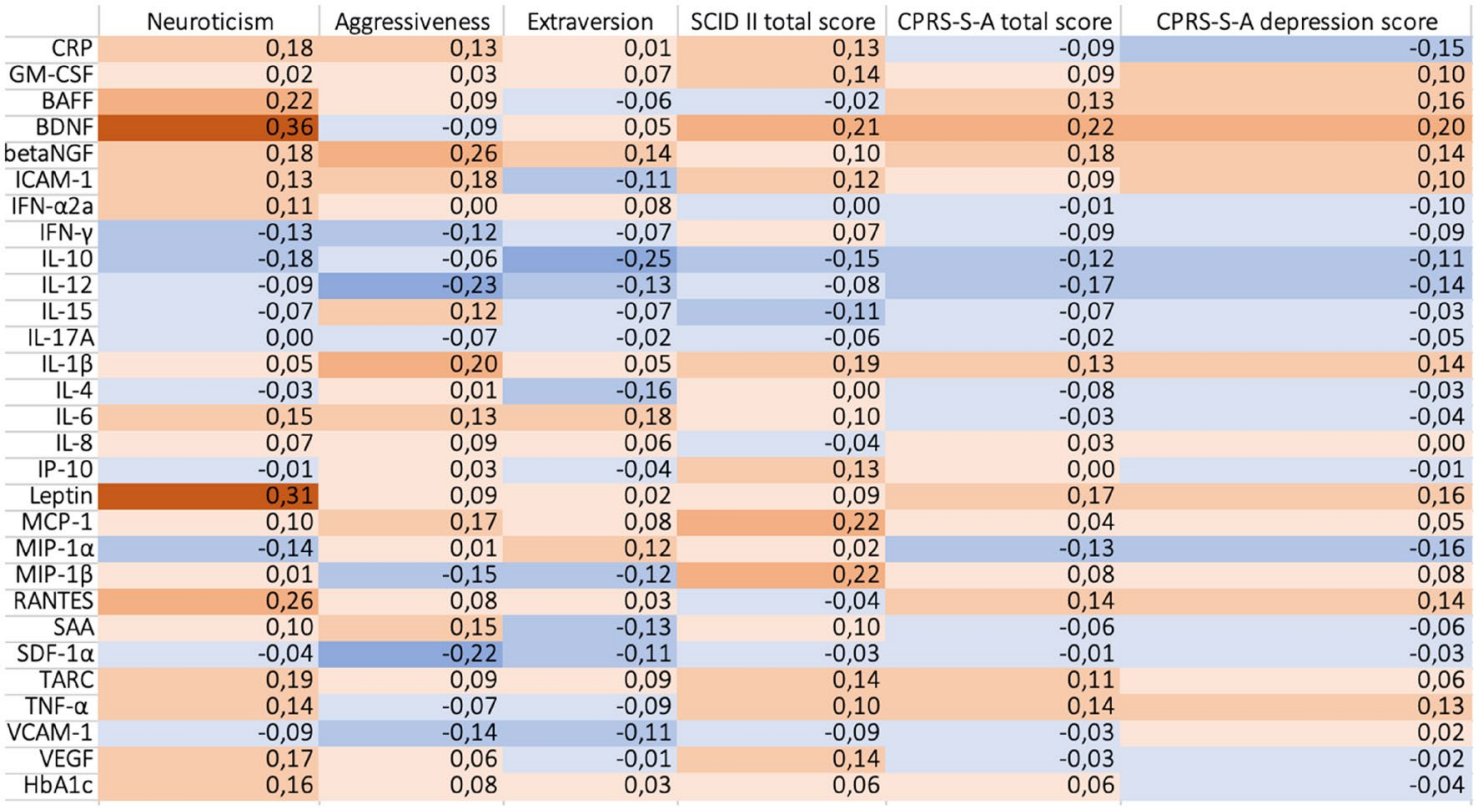
Correlation heatmap illustrating Spearman’s coefficients between biomarkers and Swedish Universities Scales of Personality, Structured Clinical Interview for DSM-IV Axis II Disorders (SCID II), Comprehensive Psychopathological Rating Scale-Self rating for-Affective disorders (CPRS-S-A) total score, and CPRS-S-A depression score, for n = 54 participants. Positive correlations in orange and negative correlations in blue. *Note*: CRP: C-reactive protein, GM-CSF: Granulocyte–macrophage colony-stimulating factor, BAFF: B-cell activating factor, BDNF: Brain-derived neurotrophic factor, betaNGF: beta nerve growth factor, ICAM-1: Intercellular adhesion molecule 1, IFN- α2a: interferon alfa2-a, IFN-γ: Interferon gamma, IL-10: interleukin 10, IL-12: Interleukin 12, IL-15: interleukin 15, IL-17A: Interleukin 17A, IL-1 β: Interleukin 1 beta, IL-4: Interleukin 4, IL-6: Interleukin 6, IL-8: Interleukin 8, IP-10: Interferon-gamma induced protein 10, MCP-1: Monocyte chemoattractant protein 1, MIP-1α: Macrophage inflammatory protein-1 alpha, MIP-1β: Macrophage inflammatory protein 1 beta, RANTES: Regulated upon activation, normal T Cell expressed and presumably secreted, SAA: Serum amyloid A, SDF-1 α: Stromal cell-derived factor 1 alpha, TARC: Thymus and activation regulated chemokine, TNF-α: Tumor necrosis factor alpha, VCAM-1: Vascular cell adhesion molecule 1, VEGF: Vascular endothelial growth factor, HbA1c: Hemoglobin A1c.

For the exploratory analysis, a cut-off for correlation was determined as a Spearman’s correlation coefficient ρ ≥ 0.30. A positive correlation above cut-off was found between trait neuroticism and BDNF (ρ = 0.36, *p* < 0.01). There were no correlations above cut-off with any of the markers for extraversion or aggressiveness. For a full description of correlations, see Fig. 1. As seen in Fig. 1, there were no correlations above cut-off between CPRS-S-A score, CPRS-S-A depression score, and any of the biomarkers. For a description of CPRS-S-A items in relation to biomarkers, see supplementary Fig. S1. There were no correlations above cut-off between SCID-II total score and any of the biomarkers, as can be seen in Fig. 1. Nor was there any significant difference in markers between participants with and without PD in 2016, all *p* values > 0.05.

GLzMs were then used to further investigate correlations in cases where ρ ≥ 0.30. A regression analysis for BDNF and neuroticism revealed an OR of 1.02 (95% CI 1.01; 1.04, *p* < 0.01) in the crude model. When adjusting for BMI, current antidepressant treatment, and sex, the OR was 1.02 (95% CI 1.01; 1.04, *p* < 0.01), which on average corresponded to an increase in BDNF of 19 pg/ml per unit increase in neuroticism score. An illustration of neuroticism scores in relation to levels of leptin and BDNF can be seen in supplementary Fig. S2.

We then examined biomarkers in relation to trajectories of personality traits over time. The RCI analysis revealed six participants with reliable decrease, and none with reliable increase in neuroticism, at follow-up. The participants with reliable decrease showed lower levels of leptin than the group which did not decrease in neuroticism, se supplementary Fig. S3. However, a Mann–Whitney test showed that the difference was not significant (z = 1.54, *p* = 0.12). Regarding aggressiveness, there was one participant with reliable decrease and one participant with reliable increase. For extraversion, there was one participant with reliable decrease and three participants with reliable increase.

A decrease in SSP neuroticism score from baseline to follow-up was negatively correlated with beta- Nerve growth factor (bNGF) (ρ = − 0.32, *p* = 0.03) and leptin (ρ = − 0.38, *p* = 0.01). No biomarker had a correlation with changes in trait aggressiveness between the two timepoints. Increases in trait extraversion between the two timepoints had negative correlations with markers IL-10 and vascular cellular adhesion molecule (VCAM) (ρ = − 0.47, *p* < 0.001 and ρ = − 0.37, *p* < 0.01, respectively).

Leptin values were not correlated above cut-off with BDNF, IL-10 bNGF, nor VCAM so these likely represent separately regulated biological mechanisms.

## Discussion

The main analysis in the study confirmed they hypothesis that trait neuroticism is linked to elevated levels of leptin. The exploratory analysis found a positive correlation, independent from leptin, between trait neuroticism and BDNF. No correlations between immunometabolic markers and PDs, assessed both categorically and dimensionally, were identified.

The finding that neuroticism and leptin had a correlation was robust when possible confounders were controlled for including sex and medications with metabolic side effects. The mean BMI of participants was overweight, as defined by the World Health Organization. The level of leptin in blood is closely correlated with body fat^41^. In our analyses, BMI was highly correlated with leptin (ρ = 0.78) and therefore not adjusted for. Leptin might act as a proxy for the association between BMI and neuroticism (ρ = 0.62, *p* < 0.001) in our results, but this is questionable in relation to earlier research showing variable metabolic activity within the same BMI range^42^. The correlation between high neuroticism and elevated leptin levels was in line with our hypothesis as well as with a previous study of another cohort of young adults published by Syk et al.^11^. In that study, the effect size expressed as ln(β) was 0.019 for the estimate of neuroticism predicting leptin, which is similar to the finding in our study (OR 1.06). The study by Syk et al. assessed a population similar to the population in the current study in regards to sex. The effect size reflects one unit change in SSP neuroticism score, which is small from a clinical perspective. A more clinically valid way to express effect size could be to use a ten-point change in SSP neuroticism score, which would render an OR of 1.79.

Leptin has been suggested to be a factor in the development of metabolic disorders such as diabetes, and might act as an early marker of metabolic disorder or complications^43^. In our study, there was no correlation above cut-off between any personality trait and HbA1c, but leptin and HbA1c correlated. This might be indicative of leptin being an early marker of metabolic dysregulation, but as it was outside the scope of the current study this was not explored further. Neuroticism has been found to be associated with an increased risk of the metabolic syndrome^21,22^. The association between elevated leptin and neuroticism in our cohort might be an early sign of this development. If neuroticism is causative of raised blood leptin, this would call for early interventions for this personality trait in order to limit deleterious long-term somatic effects. There is some evidence of metabolic syndrome being more common in borderline PD^44,45^, but knowledge of other PDs is limited. In our study, there was no correlation between PD assessed both dimensionally and categorically, and leptin. Future studies of PD and the metabolic syndrome are called for.

In our study, neuroticism also had a positive correlation with BDNF in peripheral blood. This was an unexpected finding that was unrelated to the leptin levels. A previous study showed an inverse relationship between BDNF and neuroticism in serum, but no relationship between BDNF and neuroticism in plasma^46^, whereas another study found low plasma BDNF was associated with depressive personality traits^47^. Our finding could not be explained by current antidepressant treatment (Supplementary Fig. S4), as previous publications have suggested^48,49^. Narratives are emerging that longer duration of depression is correlated with higher levels of BDNF^50^. Our cohort was a follow-up study of participants who all had psychiatric disorders at baseline and the high levels of BDNF may have been found among those with ongoing symptoms since baseline, but this was not explored further.

The DSM-5 states that PDs are categorical constructs, but in section III also presents an alternative model of PD, as a suggestion for future research^6^. In light of this, the study included both PD diagnosis and SCID-II score in an attempt to view the PD construct in both categorical and dimensional manners but did not find any correlations with any of the assessed biomarkers. Since PDs are associated with an increased risk of a number of physical conditions and disorders where inflammation is known to play a role, such as cardiovascular disorders^51,52^, it could be suspected that inflammatory system activation in PD plays a role in their development. However, PDs are a heterogenic syndrome group, and the categorical construct is coarse. This makes it hard to find significant results in small samples, such as those in the current study.

There were only seven males in the cohort. There were no significant differences in SSP dimensions between females and males. The correlations between personality traits and immunometabolic markers remained even after adjusting for sex. Some previous studies have shown that personality and immunological markers correlate differently between sexes^14^, but this could not be shown in the current study.

In the analyses of immunometabolic markers in relation to trajectories of personality traits over time, the participants with the highest leptin values were found within the group with persistent neuroticism between the two timepoints (see Supplementary Fig. S3). No participant had an RCI increase in neuroticism over time, meaning that this trajectory could not be investigated further. Furthermore, as there were no data on leptin levels at baseline, we could not draw conclusions on causality. However, the association between leptin and neuroticism is clear in data from a cohort from the same catchment population at a timepoint that corresponds to the baseline in our study^11^. It would be interesting to see if leptin is a prognostic factor for sustained neuroticism over time, which is a suggestion for future research.

There were several limitations to our study. First, the small sample size (n = 54) must be addressed. The drop-out analysis revealed a few significant differences between participants and those lost to follow-up (n = 144) regarding presence of substance abuse and in the personality trait extraversion, but not in other psychiatric disorders, personality trait dimensions, or total SCID II score, meaning that the participants were representative of the original cohort in many aspects. However, limited effect sizes in small samples may contribute to statistical errors. The participants in the cohort were thoroughly assessed at both baseline and follow-up. The thorough diagnostic assessment was considered to partly make up for the small sample size. The psychiatric assessments were performed with the LEAD procedure, which is considered the gold standard for psychiatric diagnostic assessments, and is recommended by the Swedish Health Technology Institute^53^. Another limitation concerns the construct validity of the SSP three-factor solution used in the study. The three-factor solution was based on the original work by Gustavsson et al.^35^, where factor analysis rendered the three personality dimensions (aggressiveness, extraversion, and neuroticism). These have been found to correlate to other personality inventories, such as the revised NEO personality inventory (NEO-PI-R)^54^. However, there are conflicting views on how traits and dimensions correlate to each other, and consequences for construct validity of the SSP.

Due to the study design, it was not possible to assess personality or PDs at a separate time from the assessment of other psychiatric disorders at follow-up, meaning that an individual who suffered from major depression at the assessment could be evaluated for PDs at the same timepoint. There is a concern when studying personality traits and PD that an individual’s current mental state will affect their reports on their own personality, which is often thought of as an aspect of the “state-trait issue”^55^. Clinically, the issue is often handled through treating clinical syndromes before assessing personality and PDs. To address the state-trait issue in the current study, CPRS-S-A was filled out by participants. This revealed a state issue, but the CPRS-S-A scores were moderate. The mean score in the cohort was 12.9 (SD 8.1), with the maximum possible CPRS-S-A score being 57, which at least partly supports there being less of a state effect in the cohort. Furthermore, as there was a discrepancy in the findings, with no correlations above cut-off between CPRS-S-A score and any of the biomarkers (for a full description of correlations between CPRS-S-A items and biomarkers, see supplementary Fig. S1), this might indicate that personality traits as measured in the study were not state effects, but actual personality traits. Another limitation concerning the sample size was that two of the analyzed markers, granulocyte–macrophage colony-stimulating factor (GM-CSF) and IL-4, had few samples with values above LLOD (see Supplementary table S1), which limited the information on these. The ranges of GM-CSF and IL-4 in the samples were close to LLOD, which may have decreased the accuracy of these measures. Another limitation to the study concerns the finding that there were no participants with a cluster A PD, two participants with cluster B PD, and 17 participants with cluster C PD. The generalizability of results to cluster A and B PDs is therefore limited.

The major strengths of the study were the use of gold standard psychiatric diagnostics, with excellent IRs between raters and the exclusive use of validated instruments, as well as the long follow-up time, which enabled us to control for stability of personality traits and PDs. The self-rating instruments were chosen carefully. The SSP was chosen as the personality inventory for the study based on its good psychometric properties and validity for Swedish populations^35^, and because it has been shown to correspond to other major personality models^56^. The CPRS-S-A was chosen based on its good psychometric properties^57^.

## Conclusion

The findings of the study suggest that personality trait neuroticism is correlated with higher leptin levels in peripheral blood in young adult former psychiatric patients followed up after 12 years, in line with previous findings in a similar cohort. Results indicate that investigation of the combination of persistent neuroticism over time with elevated leptin as a risk factor for development of metabolic syndrome would be relevant for future research.

### Supplementary Information

Supplementary Information.

## Data Availability

Data from the study are available from the corresponding author upon request, for the purposes of evaluating the manuscript, in accordance with the General Data Protection Regulation.
