# Supplementary material for: Correlations between personality traits, personality disorders, and immunometabolic markers
Source: Sci Rep. 2024 May 21;14:11635. doi: 10.1038/s41598-024-62214-9 (PMC11109176; doi:10.1038/s41598-024-62214-9)
Supplement: Supplementary file 1 — Supplementary Information. [file 41598_2024_62214_MOESM1_ESM.docx]

*Supplementary Table S1: Distribution of biomarkers, lowest level of detection (LLOD), numbers above LLOD, and coefficients of variation in blood samples from cohort (n = 54).*

| Biomarker (pg/ml) | Median (25 %ile; 75 %ile) | Mean LLOD | Number above LLOD, n (%) | Coefficient of variation (%) |
| --- | --- | --- | --- | --- |
| CRP | 925,702 (277,304; 2,486,975) | 3,362 | 54 (100) | 2.5 |
| GM-CSF | 0.02 (0.02; 0.24) | 0.20 | 17 (32) | 5.8 |
| BAFF | 700 (607; 857) | 0.24 | 54 (100) | 2.5 |
| BDNF | 678 (468; 1,394) | 12.33 | 54 (100) | 4.0 |
| betaNGF | 1.86 (1.47; 2.25) | 0.14 | 54 (100) | 4.5 |
| ICAM-1 | 366,115 (336,583; 426,832) | 1,907 | 54 (100) | 6.3 |
| IFN-α2a | 1.29 (0.72; 2.17) | 0.60 | 42 (78) | 3.4 |
| IFN-γ | 4.97 (3.76; 9.16) | 0.60 | 54 (100) | 3.5 |
| IL-10 | 0.26 (0.18; 0.45) | 0.13 | 51 (94) | 3.3 |
| IL-12/IL-23p40 | 164 (117; 216) | 0.29 | 54 (100) | 4.7 |
| IL-15 | 2.14 (1.81; 2.48) | 0.15 | 54 (100) | 4.0 |
| IL-17A | 2.30 (1.88; 3.20) | 0.33 | 54 (100) | 6.8 |
| IL-1β | 0.03 (0.01; 0.12) | 0.09 | 27 (50) | 3.6 |
| IL-4 | 0.00 (0.00; 0.00) | 0.03 | 9 (17) | 3.3 |
| IL-6 | 0.55 (0.41; 0.97) | 0.14 | 54 (100) | 2.2 |
| IL-8 | 3.08 (2.49; 4.01) | 0.13 | 54 (100) | 2.9 |
| IP-10 | 308.60 (225.8; 424.8) | 0.52 | 54 (100) | 4.8 |
| Leptin | 3,194 (1,099; 12,651) | 24.59 | 54 (100) | 5.5 |
| MCP-1 | 56.98 (48.96; 68.22) | 0.17 | 54 (100) | 4.8 |
| MIP-1α | 6.62 (1.18; 14.09) | 11.82 | 27 (50) | 6.6 |
| MIP-1β | 45.90 (37.20; 56.49) | 1.22 | 54 (100) | 3.0 |
| RANTES | 88,152 (42,568; 158,228) | 65.30 | 54 (100) | 2.4 |
| SAA | 2,603,648  (1,560,456; 5,005,112) | 11,856 | 54 (100) | 6.1 |
| SDF-1α | 1416 (1,172; 1,807) | 189.10 | 54 (100) | 5.3 |
| TARC | 73.85 (47.75; 115.24) | 0.43 | 54 (100) | 3.6 |
| TNF-α | 2.04 (1.71; 2.48) | 0.26 | 54 (100) | 4.1 |
| VCAM-1 | 414,701 (367,730; 478,440) | 6,765 | 54 (100) | 3.5 |
| VEGF | 28.7 (20.6; 38.7) | 0.51 | 54 (100) | 5.0 |
| HbA1c (n = 53) mmol/mol | 32.00 (30.00; 34.00) |  |  | < 4 |

CRP: C-reactive protein, GM-CSF: Granulocyte-macrophage colony-stimulating factor, BAFF: B-cell activating factor, BDNF: Brain-derived neurotrophic factor, betaNGF: beta nerve growth factor, ICAM-1: Intercellular adhesion molecule 1, IFN- α2a: interferon alfa2-a, IFN-γ: Interferon gamma, IL-10: interleukin 10, IL-12: Interleukin 12, IL-15: interleukin 15, IL-17A: Interleukin 17A, IL-1 β: Interleukin 1 beta, IL-4: Interleukin 4, IL-6: Interleukin 6, IL-8: Interleukin 8, IP-10: Interferon-gamma induced protein 10, MCP-1: Monocyte chemoattractant protein 1, MIP-1α: Macrophage inflammatory protein-1 alpha, MIP-1β: Macrophage inflammatory protein 1 beta, RANTES: Regulated upon activation, normal T Cell expressed and presumably secreted, SAA: Serum amyloid A, SDF-1 α: Stromal cell-derived factor 1 alpha, TARC: Thymus and activation regulated chemokine, TNF-α: Tumor necrosis factor alpha, VCAM-1: Vascular cell adhesion molecule 1, VEGF: Vascular endothelial growth factor, HbA1c: Hemoglobin A1c.

*Supplementary figure S1*: *Correlation heatmap illustrating Spearman’s coefficient between biomarkers and CPRS-S-A items. Positive correlations in orange and negative correlations in blue.*


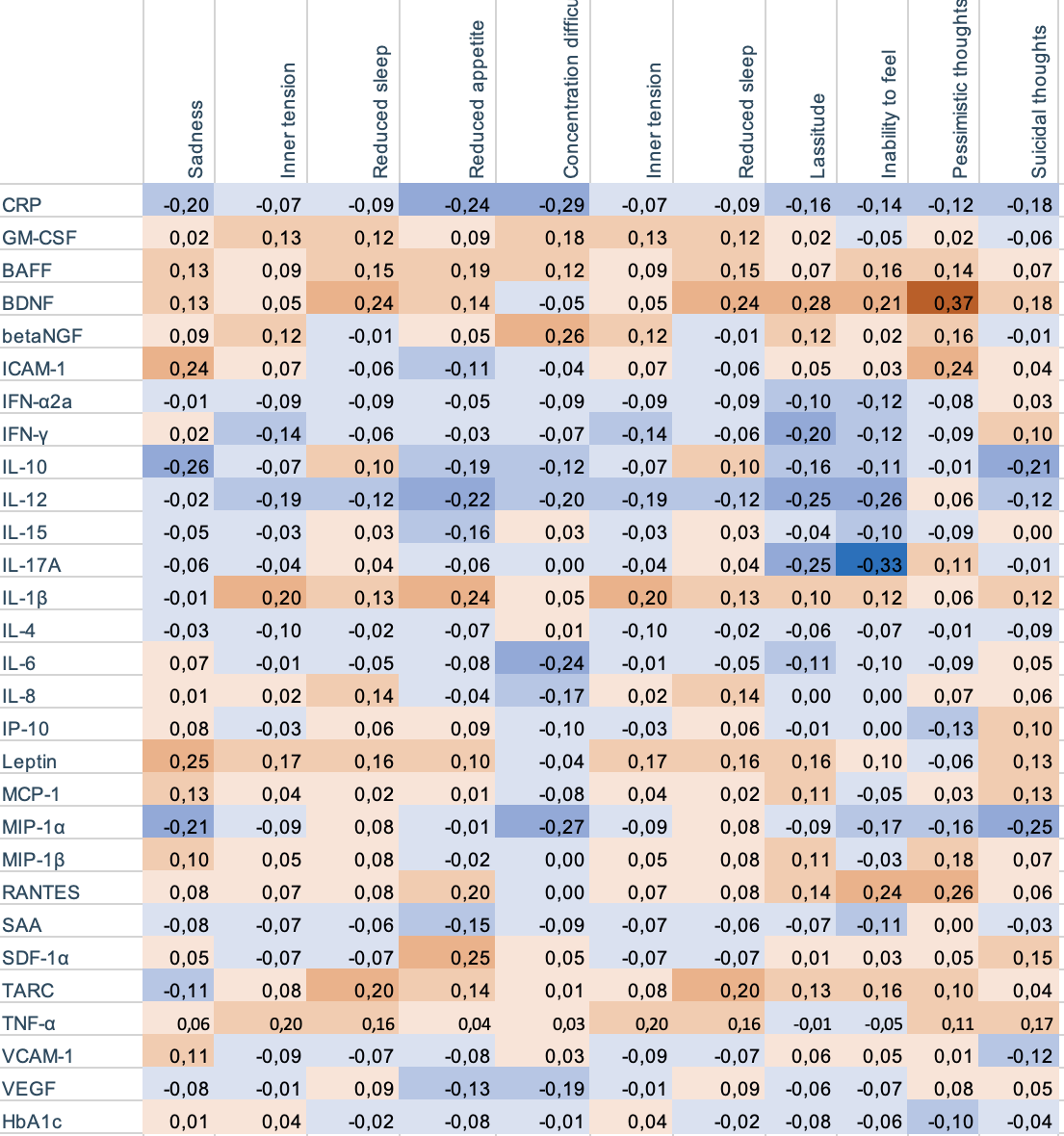


CRP: C-reactive protein, GM-CSF: Granulocyte-macrophage colony-stimulating factor, BAFF: B-cell activating factor, BDNF: Brain-derived neurotrophic factor, betaNGF: beta nerve growth factor, ICAM-1: Intercellular adhesion molecule 1, IFN- α2a: interferon alfa2-a, IFN-γ: Interferon gamma, IL-10: interleukin 10, IL-12: Interleukin 12, IL-15: interleukin 15, IL-17A: Interleukin 17A, IL-1 β: Interleukin 1 beta, IL-4: Interleukin 4, IL-6: Interleukin 6, IL-8: Interleukin 8, IP-10: Interferon-gamma induced protein 10, MCP-1: Monocyte chemoattractant protein 1, MIP-1α: Macrophage inflammatory protein-1 alpha, MIP-1β: Macrophage inflammatory protein 1 beta, RANTES: Regulated upon activation, normal T Cell expressed and presumably secreted, SAA: Serum amyloid A, SDF-1 α: Stromal cell-derived factor 1 alpha, TARC: Thymus and activation regulated chemokine, TNF-α: Tumor necrosis factor alpha, VCAM-1: Vascular cell adhesion molecule 1, VEGF: Vascular endothelial growth factor, HbA1c: Hemoglobin A1c.

*Supplementary figure S2: Participants’ neuroticism scores in relation to levels of brain derived neurotrophic factor (BDNF) and leptin.*

**

**BDNF: Brain derived neurotrophic factor

Supplementary *figure S3: Leptin values in participant males and females without and with reliable change in neuroticism between study points.*

*
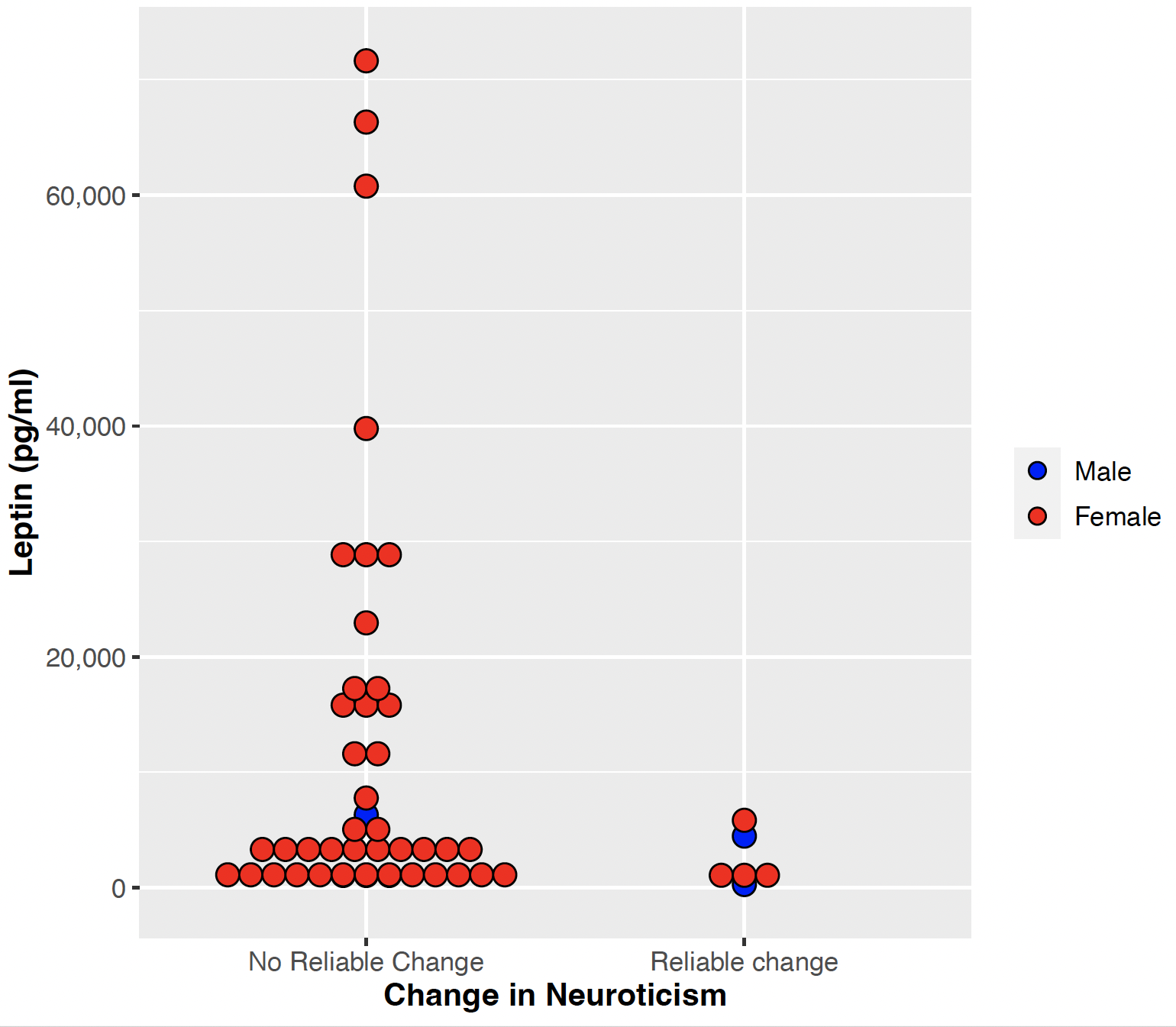
*

*Supplementary figure S4: Distribution of Brain derived neurotrophic factor (BDNF) in participants in groups without (0) and with (1) current antidepressant treatment.*

**

BDNF: Brain derived neurotrophic factor
